# DNA storage: The future direction for medical cold data storage

**DOI:** 10.1016/j.synbio.2025.03.006

**Published:** 2025-03-14

**Authors:** Peilin Shen, Yukui Zheng, CongYu Zhang, Shuo Li, Yongru Chen, Yongsong Chen, Yuchen Liu, Zhiming Cai

**Affiliations:** a Department of Urology, The First Affiliated Hospital of Shantou University Medical College, Shantou, Guangdong Province, PR China; b The First Affiliated Hospital of Shantou University Medical College, Shantou, Guangdong Province, PR China; c Shantou University Medical College, Shantou, Guangdong Province, PR China; d School of Artificial Intelligence, University of Chinese Academy of Sciences, Beijing, PR China; e BGI-Shenzhen, Shenzhen, Guangdong Province, PR China; f BGI Hospital Groups, Ltd., Shenzhen, Guangdong Province, PR China; g Department of Emergency Intensive Care Unit, The First Affiliated Hospital of Shantou University Medical College, Shantou, Guangdong Province, PR China; h Department of Endocrinology, The First Affiliated Hospital of Shantou University Medical College, Shantou, Guangdong Province, PR China; i Shenzhen Institute of Translational Medicine, Shenzhen Second People's Hospital, The First Affiliated Hospital of Shenzhen University, Health Science Center, Shenzhen University, Shenzhen, Guangdong Province, PR China; j Key Laboratory of Medical Reprogramming Technology, Shenzhen Second People's Hospital, The First Affiliated Hospital of Shenzhen University, Shenzhen, Guangdong Province, PR China; k Shenzhen Institute of Synthetic Biology, Shenzhen Institutes of Advanced Technology, Chinese Academy of Sciences, Guangdong Province, PR China; l Guangdong Key Laboratory of Systems Biology and Synthetic Biology for Urogenital Tumors, Shenzhen, Guangdong Province, PR China; m State Engineering Laboratory of Medical Key Technologies Application of Synthetic Biology, Shenzhen Second People's Hospital, The First Affiliated Hospital of Shenzhen University, Shenzhen, Guangdong Province, PR China; n Carson International Cancer Center of Shenzhen University, Shenzhen, Guangdong Province, PR China

**Keywords:** DNA storage, Medical data, Data storage, Synthetic biology, Systematic review

## Abstract

DNA storage, characterized by its durability, data density, and cost-effectiveness, is a promising solution for managing the increasing data volumes in healthcare.

This review explores state-of-the-art DNA storage technologies, and provides insights into designing a DNA storage system tailored for medical cold data. We anticipate that a practical approach for medical cold data storage will involve establishing regional, *in vitro* DNA storage centers that can serve multiple hospitals. The immediacy of DNA storage for medical data hinges on the development of novel, high-density, specialized coding methods. Established commercial techniques, such as DNA chemical synthesis and next-generation sequencing (NGS), along with mixed drying with alkaline salts and refined Polymerase Chain Reaction (PCR), potentially represent the optimal options for data writing, reading, storage, and accessing, respectively. Data security could be promised by the integration of traditional digital encryption and DNA steganography. Although breakthrough developments like artificial nucleotides and DNA nanostructures show potential, they remain in the laboratory research phase.

In conclusion, DNA storage is a viable preservation strategy for medical cold data in the near future.

## Graphical abstract

**Figure:**
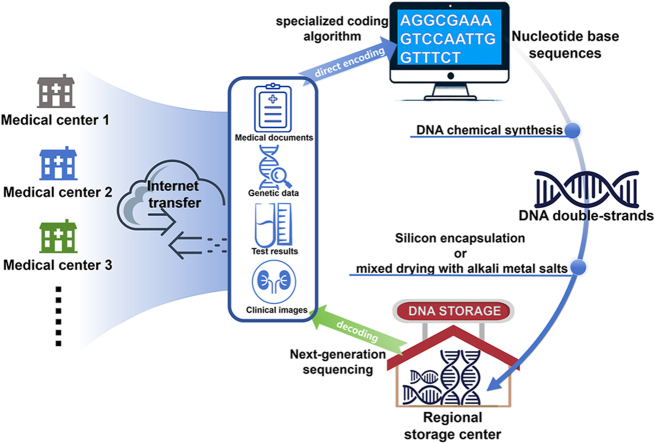

## 1 Introduction

In the current era, we are witnessing an unprecedented surge in information. Between 2010 and 2018, the number of global data center compute instances increased by 550 % [1]. The corresponding energy consumption of these data centers, reaching 200 TW-hours (TWh) in 2018, rivals that of some countries at the time [2]. The International Data Corporation (IDC) predicted a staggering growth in global data volume from 33 Zettabytes (ZB) in 2018 to 175 ZB by 2025 [3]. In 2021, IDC projected a compound annual growth rate of over 20 % for the global data volume [4], estimating a global data burden of over 435 ZB by 2030.

This formidable data challenge to the existing storage capacities is primarily attributed to the gap between rapid data expansion and sluggish advancements in storage media. On one hand, the acceleration of fields such as medical health, precision medicine, bioengineering, artificial intelligence (AI), the Internet of Everything, and 5G communication substantially contribute to large-scale data production. For example, with the rapid development of the Internet of Things (IoT), IDC estimated that about 75 % of the world's population will interact with an IoT device every 18 s, generating a cumulative data volume surpassing 90 ZB by 2025 [3]. On the other hand, current storage media are approaching their limits and struggling to meet the needs of massive data storage. Traditional storage media like hard disk drives (HDD) and solid-state drives (SSD) currently dominate the field of data storage. Despite reported progress in storage density (from 380 Gigabyte per square inch (GB/inch^2^) to 1100 GB/inch^2^ for HDD, from 200 GB/inch^2^ to 2000 GB/inch^2^ for NAND) and cost reduction (from 0.272 United States Dollars per Gigabyte (USD/GB) to 0.039 USD/GB for HDD, from 3.33 USD/GB to 0.320 USD/GB for NAND) from 2008 to 2016, they still fall short of Moore's Law predictions [5].

The healthcare industry, a paramount aspect of human well-being, is at the forefront of grappling with the limitations of current data storage. The diversity and complexity of medical results and records, along with the rapid advancements in genomics, have led to an unprecedented increase in healthcare data. In 2018, IDC estimated that healthcare data constituted 30 % of the global data volume and would reach 36 % by 2025 [3]. This growth rate surpasses that of manufacturing by 6 %, financial services by 10 %, and media and entertainment by 11 % [3]. Due to medical and legal requirements, healthcare data often necessitates long-term storage, consuming substantial resources, including physical storage space, manpower, materials, and daily maintenance. Therefore, there is an urgent need for an innovative, high-density storage medium to address the burgeoning data crisis in the field of healthcare.

Currently, the exploration of new storage media primarily revolves around two key tracks. One is silicon-based storage media, such as SSD and optical tapes [6]. The other is carbon-based storage media, encompassing specific small organic molecules [7], peptide sequences [8], synthetic metabolomes [9], and DNA. Among these options, we perceive DNA as the most promising candidate for medical data storage. DNA carries the genetic information essential to understanding the physiological and pathological conditions of every organism. Therefore, researchers have never ceased to unlock DNA's mysteries, discovering its capacity to store data using the permutation and combination of the four bases: Adenine (A), Guanine (G), Cytosine (C), and Thymine (T). This finding positions DNA not only as a substantial data source in healthcare but also as a potential solution for medical data storage.

Given the considerable prospects of DNA storage for medical data storage, we aim to review the state-of-the-art technologies related to the DNA storage workflow, explore their applications in medical data storage, and endeavor to design a DNA storage system based on existing technologies in the near future.

## 2 Resolving medical data crises with DNA storage

### 2.1 Medical data characteristics

At present, healthcare data predominantly comprise medical tests, examinations, images, and patient history records. Looking ahead, genomic data are set to add a significant burden to medical data storage. For instance, examining the genome sequences of triplets (index patient, father, and mother) as new index patients emerge in a family, and analyzing cancer genomics for prognosis prediction are becoming increasingly common. IDC's estimation indicated that the average data volume of healthcare and life science generated per person is approximately 270 GB, with the majority categorized as cold data [10], which refers to information that requires long-term storage and low access frequency. According to the Shenzhen National Gene Bank, a staggering 80 % of current data falls into the category of cold data [11]. This proportion is anticipated to be even higher in the healthcare sector.

Medical cold data is complex and enormous. After a single treatment course, extensive text data (such as historical medical records, diagnostic reports, and laboratory results), image data (including X-rays, MRIs, or CT scans), and genomic data are generated. Currently, we use past medical data as a control to compare against new medical results to detect changes in a disease, such as the morphological changes in a tumor. With advancements in DNA sequencing technology, we can now store Illumina sequencing slides in freezers, allowing for re-sequencing whenever necessary. These data are stored as medical cold data, often with infrequent or no access. Despite this, they must be preserved over an extended period for regulatory compliance and historical archiving. Using traditional storage media such as paper and hard drives, medical cold data storage not only requires extensive physical space but also incurs significant maintenance costs. Despite these investments, the data preserved on such media is prone to degradation, with a lifespan limited to mere decades. The limited durability of current storage media partly accounts for the short medical data retention mandates—6 years in the USA [12] and 15 years in China [13]. From our perspective, ideally, medical data retention should extend over the lifetimes of the individuals concerned. For instance, the National Health Service (NHS) of the United Kingdom stipulates a mandatory medical record retention period of 10 years after a patient's demise [14]. Furthermore, anticipating future needs for scientific research and data analysis further highlights the importance of extending medical data retention periods.

### 2.2 An overview of DNA storage technology

DNA storage emerges as an appealing solution for future medical data storage owing to its low energy consumption, high data density, and the capability for multiple and random access. The inherent properties of DNA storage match the needs for storing extensive medical data well, presenting a promising approach to meet the challenges and regulatory requirements of healthcare information.

**Fig. 1:**
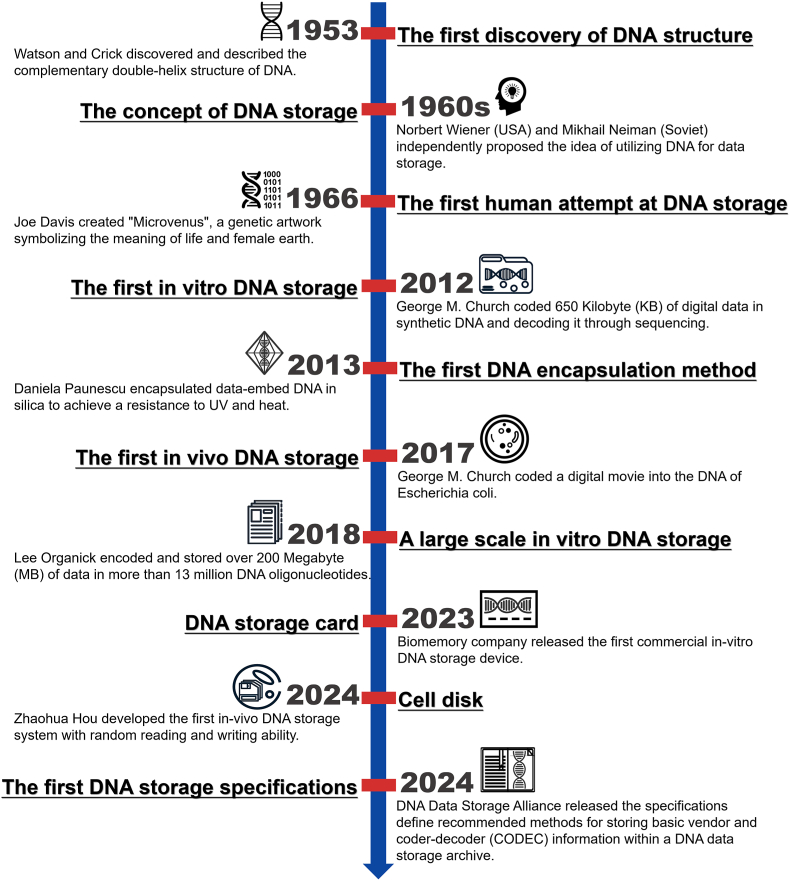
Historical milestones in the development of DNA storage.

In 1953, Watson and Crick first discovered and described the complementary double-helix structure of DNA [15]. In the 1960s, alongside the release of IBM's first hard disk, Norbert Wiener (USA) and Mikhail Neiman (Soviet) independently proposed the idea of utilizing DNA for data storage almost simultaneously. Later, a significant milestone was reached in 1966 when Joe Davis created "Microvenus", a genetic artwork symbolizing the meaning of life and female earth, marking the first human attempt at DNA storage [16]. Over the past few decades, substantial progress in DNA synthesis and sequencing has catalyzed breakthroughs in DNA storage. In 2012, Church et al. from Harvard Medical School achieved the first *in vitro* DNA storage application, coding 650 Kilobyte (KB) of digital data in synthetic DNA and decoding it through sequencing [17]. Furthering their pioneering work, the same team made another breakthrough in 2017 by using the CRISPR/Cas system to encode a digital movie into the genomes of *Escherichia coli* [18]. To enhance data integrity in DNA storage, Paunescu et al. were the first to encapsulate data-embedded DNA in silica, significantly improving its resistance to UV radiation and heat in 2013 [19]. In 2018, Organick et al. further advanced *in vitro* DNA storage capacity, successfully storing and retrieving over 200 Megabytes (MB) of data using 13 million DNA oligonucleotides [20]. In 2023, we witnessed the launch of the first commercial *in vitro* DNA storage device—a 1000 USD DNA storage card capable of storing 1 KB of data [21]. It is designed to preserve precious text with unyielding reliability, maximum security, eco-friendliness, and timeless compatibility. Last year, Hou et al. further optimized the CRISPR/Cas system and developed the "Cell Disk", the first *in vivo* DNA storage system capable of random reading and rewriting as flexibly as modern hard drives [22]. Most excitingly, on March 12th, 2024, the DNA Data Storage Alliance, which includes major industry players like Illumina, Microsoft, and Twist Bioscience, introduced the first DNA data storage specifications [23]. The specifications are structured around two main components: Sector Zero and Sector One. Sector Zero contains essential information to identify the vendor responsible for synthesizing the DNA and the Coder-Decoder (CODEC) used for coding the data. This sector ensures that any DNA storage system can recognize the manufacturing company and the coding method, facilitating compatibility across different systems. Sector One includes metadata about the contents, a file table, and parameters required for data transfer to a sequencer. This design ensures that DNA-stored data can be effectively accessed and read, standardizing the retrieval process. The specifications aim to foster an interoperable DNA data storage ecosystem, facilitating cooperation among various systems and paving the way for broader commercialization of DNA-based data storage solutions. These historical milestones in the development of DNA storage were shown in Fig. 1.

**Fig. 2:**
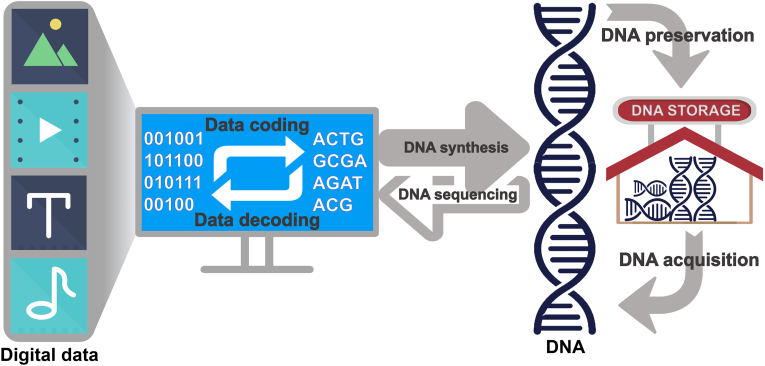
**Illustration of the DNA storage workflow.** Digital data, such as images, videos, texts, and audio, is encoded into binary code, represented by "0" and "1"; and then converted into nucleotide sequences, including Adenine (A), Guanine (G), Cytosine (C), and Thymine (T). Subsequently, these nucleotide sequences are synthesized into DNA molecules for storage. To retrieve the original digital data, the data-embedded DNA is extracted, sequenced, and converted back into binary code for computer recognition.

The fundamental principle of DNA storage involves converting digital data into nucleotide sequences by coding the binary digits "0" and "1" from computers into DNA bases (A, T, C, G) during synthesis. The synthesized DNA is then stored under specific conditions for long-term preservation. Subsequently, DNA sequencing technology is used to read the stored sequences, allowing the data to be decoded back into its original digital format for computer recognition. At present, the basic workflow of DNA storage has been established, including six processes: data coding, DNA synthesis, DNA preservation, DNA acquisition, DNA sequencing, and data decoding (Fig. 2). Although several research teams have successfully designed DNA storage systems capable of executing the entire workflow, these systems remain far from practical application [24,25].

Despite the theoretical potential for a gram of single-stranded DNA (ssDNA) to store up to 0.455 ZB of data [26] lasting thousands of years [27], DNA storage still encounters several technical constraints. First and foremost, even with technological advancements reducing costs and shortening timelines, the high cost and slow processing speeds of DNA synthesis and sequencing still present major barriers to their broad application. Secondly, being based on chemical reactions, data access and retrieval in DNA storage systems are significantly more complex and time-consuming compared to traditional computing environments. Lastly, although current error correction mechanisms allow for accurate data retrieval, inherent limitations in DNA synthesis and sequencing can introduce significant errors, complicating the employed coding strategies.

### 2.3 Prospects of DNA storage for medical cold data

The above constraints render DNA storage technology less suitable for hot data requiring frequent, instant, and precise manipulation. However, we consider that DNA storage holds the potential to surmount these limitations and find application in the medical cold data storage within the scope of future hospital informatization. In the field of healthcare, medical cold data necessitates exceptionally prolonged retention times. Consequently, the cost of DNA storage can be amortized over time. The storage process of medical cold data typically involves a one-time writing process followed by exceedingly infrequent reading, diminishing the necessity for fast processing and rendering the slow writing, accessing, and reading challenges more tolerable in this context. Additionally, a substantial volume of genomic data in the future can be directly stored in its original DNA form, eliminating the cost associated with data conversion and DNA synthesis.

Given the advantages of DNA storage for medical cold data, we anticipate the first large-scale application of DNA storage systems to be in this field. The specific requirements of medical cold data align well with the strengths of DNA storage, making it a promising solution for the unique challenges of extended retention and infrequent access typical in healthcare information.

## 3 Specific considerations for medical cold data DNA storage

### 3.1 *In vitro* vs. *in vivo* DNA storage

Despite the impressive stability of a 521-year half-life, DNA remains susceptible to damage from physical, chemical, and biological factors, leading to potential data loss [28]. Consequently, proper safeguards are imperative for DNA data storage. DNA can be preserved *in vitro*, within artificial non-living media, or *in vivo*, within living cells. Currently, *in vitro* DNA storage offers easier management, simpler coding, and quicker data writing and reading than the *in vivo* approach. However, the associated costs of synthesis and daily maintenance are higher. Conversely, *in vivo* DNA storage leverages cellular replication, transmission, and self-repair capabilities, facilitating efficient information amplification and maintenance with minimal effort and cost. Furthermore, utilizing gene editing technologies to manage bases avoids the need for *de novo* DNA synthesis, potentially enabling more cost-effective data writing within cells. However, maintaining cell survival to avoid data loss is an inevitable consideration for *in vivo* DNA storage. For medical cold data requiring lifetime retention, highly durable and economically feasible DNA preservation methods are essential. Therefore, we consider a preservation approach lasting for more than 100 years at room temperature to be suitable.

#### 3.1.1 In vitro DNA storage

Common *in vitro* DNA preservation methods, such as solution and dry powder storage, necessitate cryopreservation, not only consume significant energy but also risk compromising DNA molecule integrity. Therefore, the retention period is limited to years or decades. For medical cold data, more durable *in vitro* DNA storage methods at room temperature, such as silicon-based encapsulation and mixed drying with alkaline salts, provide more appropriate alternatives.

Inspired by ancient DNA recovered from avian eggshell fossils, where the DNA specimen was separated and protected from the environment by a dense diffusion layer [29], researchers have developed encapsulation techniques for DNA storage. DNA simply encapsulated into silica particles (SiO2) demonstrated resistance to harsh environmental conditions, enduring temperatures up to 200 °C [19]. For further improvement, Paunescu et al. designed a double-layer shell by coating TiO2 layers onto SiO2 particles, providing increased protection for the DNA core, and increasing the DNA's UV resistance by 42 times, comparable to the increase during bacterial spore formation [30]. Chen et al. embedded the multilayered nanoparticles formed by DNA and polyethyleneimine (PEI) into a protective silica layer to achieve a 7.8 wt% DNA weight load and a half-life at 20 °C ranging from 20 to 90 years [31]. Julian Koch proposed a concept of "DNA-of-Things" (DoT) [32]. In the DoT framework, data-embedded DNA molecules are encapsulated in nanoscale silicon beads and incorporated into a variety of materials for printing or casting objects of any shape. It was demonstrated that this biodegradable organosilica-encapsulated DNA remains stable at room temperature for over 60 years [33]. With further optimization, it may reach 100 years. Grass et al. encoded 83 KB of information into 4991 DNA fragments of 158 nucleotides and stored it in silica [27]. Their experiments showed that the original information can be recovered without error even after being processed for a week at 70 °C, which is equivalent to 2000 years at 9.4 °C. To further enhance durability, Coudy et al. developed an 18 mm × 7 mm, weighing 1.3 g, stainless-steel capsule named DNAshell [34]. A DNA fragment of 150-nucleotide aliquoted in glass inserts and encapsulated in the DNA shell was estimated to have a 38,000-year half-life. Nevertheless, encapsulation methods suffer from low density (usually less than 10 wt%) and high manipulation difficulty. To tackle these challenges, researchers developed a method involving the mixed drying of DNA with earth alkaline salts [35]. By forming stable internal chemical bonds, this approach has resulted in densities surpassing 30 wt%, with the estimated half-lives of the stored data ranging between 109 and 753 years.

#### 3.1.2 In vivo DNA storage

Living cells, regarded as not only the smallest biological units but also data carriers for DNA storage, present intriguing possibilities. Researchers have synthesized bacterial genomes to control cellular activity [36] and engineered yeast artificial chromosomes (YACs) for coding images and videos [37]. Utilizing the replication, passage, and DNA self-repair abilities of cells, DNA, along with its data, can be efficiently amplified and preserved for generations at a low cost. Mass production is economically viable as living cells constantly renew DNA sources. Hao et al. used a mixed culture of bacterial cells to enable an economic DNA storage on a large scale [38]. To bypass *de novo* DNA synthesis and achieve cost-effective DNA writing, researchers have adopted gene editing as a means of data writing in DNA storage following the development of the precise single-base editor (C to T or G to A) by David Liu and colleagues [39]. George M. Church demonstrated the coding of a digital movie into the genomes of living bacteria using the CRISPR/CAS gene editing system, achieving the first attempt at *in vivo* DNA storage [18]. Sadremomtaz et al. Used a CRISPR base editor to create point mutations as signals in DNA tapes to record digital data [40]. Liu et al. designed a dual-plasmid system to store and rewrite DNA information in *E. coli* based on the CRISPR/Cas12a system [41]. Hou et al. optimized the CRISPR/Cas9 system and developed the "Cell Disk", where each yeast cell functions as a separate "disk block", allowing for independent data manipulation in different cells [22]. In 2019, Liu et al. enhanced the CRISPR/Cas gene editing system by developing a prime editing (PE) system capable of introducing all 12 types of point mutations without double-strand breaks or donor DNA templates in living cells [42]. Building on the PE system, Choi et al. introduced the "DNA Typewriter" in 2022 [43]. The DNA Typewriter achieved sequential and unidirectional information recording in DNA, successfully demonstrating the capability to record thousands of symbols, complex event histories, and short text messages. Furthermore, simplification of the data writing process has been achieved by adopting gene editing technologies controlled by light [44], electric [45], and chemical signals [46]. However, the inherent vulnerability of cells poses significant concerns. The survival of cells is crucial for maintaining the integrity of data. Liu et al. constructed artificial chromosomes in engineered spore-forming Bacillus to store data in DNA with high precision and random access ability [47]. Owing to the remarkable resilience of bacillus spores, DNA encoded with data exhibits an impressive capacity to resist environmental stress such as high temperature, oxidative stress, and ultraviolet exposure. Sun et al. stored data in the genomic DNA of Halomonas bluephagenesis, an extreme halophilic bacteria that can survive and thrive in highly saline environments [48]. The genetic stability and ability to survive harsh environments make it an ideal biological data storage medium. Then, to recover data after many generations of cells, Song et al. proposed a self-error-detecting, three-base block coding scheme (SED3B) that adds error-detecting bases in small data blocks, which can combine with the inherent redundancy of DNA molecules for effective error correction [49]. Using error-prone PCR, the information encoded in *E. coli* cells is estimated to remain reliable for more than 12,000 years of continuous replication.

Both *in vitro* and *in vivo* approaches possess merits and drawbacks for medical cold data storage. However, due to the significant technological differences between *in vitro* and *in vivo* approaches, *in vitro* methods exhibit more prominent advantages in many aspects for medical cold data storage. For instance, *in vitro* storage achieves higher data density and allows for the synthesis and preservation of longer DNA molecules. In contrast, *in vivo* storage is limited by cellular DNA replication and genome stability constraints, typically accommodating shorter DNA fragments. Furthermore, living cells are susceptible to environmental stress, mutations, and cell death, leading to data loss, making their durability far inferior to that of *in vitro* storage. In terms of data manipulation, *in vitro* storage demonstrates greater maturity and accessibility. Established techniques such as PCR enable precise and efficient amplification of specific DNA fragments, facilitating reliable data operations. On the contrary, *in vivo* storage requires the use of advanced gene editing technologies, such as CRISPR/Cas systems, to manipulate DNA data, which involves higher technical complexity and potential off-target effects. Furthermore, *in vitro* DNA storage methods benefit from ongoing advancements in synthetic biology and materials science, which continue to improve the efficiency and cost-effectiveness of DNA storage. Recent developments in microfluidics and automated DNA synthesis systems have streamlined the writing, reading, and preservation processes, which are essential for handling large volumes of medical data. The introduction of silicon-based encapsulation and mixed drying with alkaline salts has demonstrated the potential to extend DNA storage lifespan to over a hundred years, making it particularly well-suited for medical cold data that requires minimal access but long-term retention. Despite the challenges faced by *in vivo* storage, its unique characteristics cannot be overlooked. For instance, *in vivo* storage leverages the self-replication and repair capabilities of living cells, theoretically enabling efficient data amplification and reducing the need for additional synthesis.

By focusing on optimizing *in vitro* methods, the healthcare industry can leverage the unique advantages of DNA storage, such as its unmatched data density and longevity, to meet the escalating demands for medical cold data storage. Unlike traditional storage media that face challenges with energy consumption and physical space limitations, DNA storage requires negligible energy for maintenance and offers unparalleled storage density, which is especially valuable in healthcare contexts with growing genomic data needs. Meanwhile, *in vivo* DNA manipulation is likely to find more active applications in the field of medicine. Although current challenges prevent it from being a viable option for large-scale cold data storage, its potential applications in continuous health monitoring and personalized medicine should not be overlooked. For example, future advancements may enable the use of *in vivo* DNA storage in implantable medical devices for recording real-time health data, which could serve as an immediate backup during emergencies or when access to external storage systems is compromised.

### 3.2 Medical data security and encryption

Both *in vitro* and *in vivo* DNA storage methods physically protect encoded data. However, like other storage methods, DNA storage still requires robust data security and encryption. Common encryption algorithms include Data Encryption Standard (DES), Advanced Encryption Standard (AES), and Rivest-Shamir-Adleman (RSA). Security measures encompass internet firewalls, digital signatures, and access control. With the increase in computing power, database breaches are becoming more frequent, making it increasingly challenging to protect confidential data.

In addition to traditional methods, DNA storage can be further safeguarded by leveraging its unique features: 1) High Data Density: Even with encryption and redundancy, DNA's data density far surpasses that of traditional technologies. 2) High Secrecy: Artificial DNA is almost indistinguishable from natural DNA, making it difficult to detect. 3) Tamper-Resistance: Mixing DNA containing confidential information with other DNA makes tampering nearly impossible. 4) Programmability: DNA storage can be combined with encryption methods to enhance data confidentiality.

In 1999, Clelland et al. encoded the phrase "JUNE 6 INVASION: NORMANDY" into about 100 base pairs (bp) of DNA and mixed it with a billion bp of "junk" DNA, demonstrating DNA's potential for information storage and encryption [50]. Since then, various DNA sequence-based encryption methods have been developed. For example, Fan et al. used mirror-image DNA strands for data storage, requiring a high-fidelity mirror-image Pfu DNA polymerase-driven PCR reaction to read the information [51]. Grass used short tandem repeat DNA sequences as an encryption key and designed a unique algorithm to ensure encryption uniqueness [52]. Li et al. concealed DNA-encoded messages within a mass of junk DNA, using a complex system of real and fake primers, with the CRISPR/Cas12a system distinguishing between them to ensure message integrity and confidentiality [53]. Chu et al. proposed a deniable encryption method that exploits DNA's inherent noise channels, utilizing two similar modulation keys to encode both real and fake messages into DNA sequences [54]. This approach makes it extremely difficult for an adversary to distinguish between true and fake data, providing a robust solution to counter coercive attacks. Similarly, Zan et al. demonstrated a method for image encryption in highly error-prone DNA storage channels [55]. Their approach leverages modulation signals and DNA sequence-based encryption techniques that are resilient to noise, insertion, deletion, and substitution errors, ensuring data integrity even with high sequencing errors. Beyond sequence-based methods, DNA structure can also store information, with changes in DNA structure recognition methods altering data reading results to achieve encryption [[56], [57], [58], [59]]. For example, Yao et al. explored a DNA structure-based encryption scheme for image data, employing DNA hybridization and gene mutation to replace image pixels and further diffuse the encrypted data [60]. This method enhances robustness against sequence loss and substitution errors, highlighting the potential of DNA structural properties in securing sensitive information.

In addition to encryption, data wiping is crucial for data security. In silicon-based systems, data is overwritten using special algorithms to make it unrecoverable. In DNA storage systems, physical and chemical destruction of DNA molecules (e.g., via UV irradiation, high temperature, or oxidizing agents) is the most direct way to erase data but requires professional equipment and often leaves residues. Therefore, DNA-specific data wiping methods have been developed. Kim et al. proposed a temperature-dependent erasure method [61]. When the temperature of the solution with true and false DNA strands rises and then returns to room temperature, the strands mix randomly, obscuring the true information. Wang et al. presented a parallel molecular computation model using DNA strand displacement reactions directly on DNA storage, enhancing data modification and enabling data erasure [62].

In the field of medicine, data security and encryption are paramount. Medical data includes names, addresses, social security numbers, and treatment records. Unauthorized disclosure of this information can breach patient privacy, leading to identity theft, discrimination, or embarrassment. Therefore, the privacy and security of medical data are protected by laws and regulations, with severe penalties for violations. Given the sensitive nature of medical data and its long-term retention requirements, DNA storage presents both opportunities and challenges in ensuring robust data security.

To ensure data security and confidentiality, hospitals implement strict access control measures, allowing only authorized personnel to access specific data. Additionally, medical data is typically encrypted during transmission and storage to prevent unauthorized access and data breaches. DNA storage inherently offers encryption capabilities. One of the unique advantages of DNA storage is the ability to leverage both its molecular properties and traditional digital security measures. The biological nature of DNA makes it inherently difficult to detect or tamper with, especially when mixed with other sequences, enhancing the secrecy of the stored information. The integration of DNA steganography with traditional encryption and security approaches provides a multi-layered defense mechanism that can achieve high security levels for medical data, ensuring compliance with stringent regulations and protecting patient privacy. DNA steganography involves concealing encoded information within seemingly random DNA sequences, making the presence of sensitive data difficult to identify. For example, medical data encoded in DNA can be embedded within genomic sequences that appear biologically plausible, further increasing security. This form of encryption offers a novel layer of protection beyond traditional cryptographic techniques. In practical terms, implementing DNA-based encryption in medical data storage involves coding the data into DNA sequences, applying both steganographic techniques and traditional encryption, and then synthesizing the encrypted DNA. The encrypted DNA can be stored in physical formats such as encapsulated silica particles or preserved in dry powder form, which are resistant to environmental factors and unauthorized access. However, additional challenges remain, such as the development of standardized protocols for secure DNA encoding and retrieval, which will require advancements in bioinformatics and cryptographic algorithms specifically designed for DNA storage. Furthermore, robust data retrieval mechanisms must be integrated to ensure that the process of decoding and extracting medical information is both efficient and secure. This may involve the use of multi-factor authentication for data access and real-time monitoring systems to detect unauthorized retrieval attempts.

In conclusion, DNA storage presents a promising avenue for enhancing medical data security by integrating its inherent molecular properties with advanced cryptographic techniques. However, further research and development are essential to address the technical and regulatory challenges, ensuring that DNA storage systems meet the stringent requirements of modern healthcare data security.

### 3.3 Specialized coding algorithms for medical data

The conventional coding method for DNA storage involves coding digital data into binary codes and then transforming it into DNA base sequences. Theoretically, one base can represent 2 bits in binary code, achieving a coding density of 2 bits per nucleotide (bits/nt). However, achieving an ideal coding density becomes challenging after introducing necessary error-correcttion codes (ECCs) and avoiding biological restrictions, including long repeat single bases, functional regions, and an unbalanced GC ratio.

Traditionally, most research efforts have focused on developing universal coding methods applicable across all data types, posing challenges to achieving significant enhancements in coding density. In 2012, Church et al. introduced the Church coding algorithm for DNA storage by simply mapping "0" to A/C and "1" to G/T, allowing for random substitution between A and C, as well as between G and T [17]. The design is based on restricted basic mapping relationships but does not adhere to biological restrictions. Other examples include the Goldman coding algorithm [26], Grass coding algorithm [27], and Blawat coding algorithm [63]. To address biological restrictions, coding algorithms with biological filtering have been developed, such as the Base64 coding algorithm [24], Fountain code [64], Yin-Yang code [65] and DNA-AEon [66]. These algorithms perform sequence filtering under biochemical constraints after basic mapping, ensuring the DNA sequence satisfies preset biochemical constraints perfectly. Additionally, to prevent data loss during DNA synthesis, preservation, and sequencing, ECCs such as Reed-Solomon Code [67], Hamming Code [68], and Raptor Code [69] are applied. Although there are many types of ECCs, most do not fully address the unique biological constraints of DNA storage, such as substitutions, deletions, and insertions of bases. Fortunately, researchers have developed various ECCs specifically tailored for DNA storage in recent years. Press et al. designed an ECC for DNA storage called Hash Encoded, Decoded by Greedy Exhaustive Search (HEDGES) [70]. HEDGES corrects DNA errors by encoding data with a hash-generated pseudo-random sequence, allowing a algorithm to detect and fix insertions, deletions, or substitutions during decoding. It converts stubborn indels into substitutions, which a backup Reed-Solomon code then repairs across strands. Tested on 3.5 % error-prone DNA, it recovers >97 % data and scales to exabyte storage with <10 % errors. Song et al. developed a de Bruijn Graph-based DNA sequence reconstruction algorithm (DBGPS) capable of recovering the original DNA sequence without error from fragments with substitutions, insertions, and deletions [71]. The error-correction mechanism breaks DNA strands into small overlapping fragments (k-mers), filters out rare/noisy fragments (likely errors), then reconstructs the original sequence by finding the most probable path in a graph. This method reliably recovers data even from heavily damaged DNA (e.g., 96.3 % success after 70 days at 70 °C) by focusing on high-confidence fragments. Besides error correction algorithms, the decoding methods for ECCs have also been improved. Ding et al. developed a soft-decision decoding software called Derrick to improve error-correcting capability in DNA digital storage [72]. Compared to traditional hard-decision strategies, Derrick doubles the error correction capability of Reed-Solomon codes and reduces the probability of uncorrectable errors by several orders of magnitude. These advanced ECCs have significantly enhanced the error correction performance of DNA storage systems, providing robust support for medical data recovery.

The above methods outline the development process of coding techniques for DNA storage (Table 1). From simple mapping to biologically constrained mapping, and from basic data error correction to specialized biological error correction, DNA storage coding process is becoming increasingly sophisticated and efficient. However, with the above methods, the coding density for DNA storage remains around 2 bits/nt. Therefore, specialized coding algorithms have been devised. Wu et al. developed an end-to-end DNA coding method for text and images, achieving logical storage densities exceeding 2 bits/nt for images and 3 bits/nt for text [73]. Notably, the example of the highest density reached 3.83 bits/nt for a 250 MB image. Furthermore, specialized DNA coding algorithms for video [74], images [[75], [76], [77]], English text [78], and Chinese text [79] have been developed. Recently, with the help of deep learning, Sun et al. proposed a more efficient paradigm for compressing digital data to DNA while excluding arbitrary sequence constraints [80]. Both standalone recurrent neural networks (RNNs) and pre-trained language models were used to extract intrinsic patterns in the data and generate probabilistic portrayals. These were then transformed into constraint-free nucleotide sequences using a hierarchical finite state machine. Utilizing these methods, a 12 %–26 % improvement in compression ratio was achieved for various data types, directly translating to up to a 26 % reduction in DNA synthesis costs. Zhang et al. utilized another neural network, convolutional neural network (CNN), to train on 6507 randomly selected images from the ImageNet database and developed a coding algorithm for those images with a density of 23.72 bits/nt—approximately 10 times higher than the theoretical density [81].

**Table 1:** Comparison of different universal DNA storage coding algorithms.

Year	Encoding algorithm	Information density (bits/nt)	Biological constrain	Error correction	Refs
2012	Church code	0.83	No	No	Church et al.
2013	Huffman code	0.33	No	Yes	Goldman et al.
2015	Reed-Solomon code	0.81	No	Yes	Grass et al.
2016	Blawat code	0.92	No	Yes	Blawat et al.
2017	Base64	1.72	Yes	Yes	Yazdi et al.
2017	Fountain code	1.57	Yes	Yes	Erlich et al.
2020	HEDGES	1.2	Yes	Yes	Press et al.
2022	DBGPS	1.3	Yes	Yes	Song et al.
2022	Yin-yang code	1.95	Yes	Yes	Ping et al.
2023	DNA-Aeon	1.17	Yes	Yes	Welzel et al.

Medical records are diverse in format, encompassing structured text, high-resolution images, and intricate genomic sequences. The volume of each type of medical data is substantial and continues to increase each year, with extended retention periods. Although universal coding algorithms can be applied to these data types, significantly increasing coding density is challenging. This challenge stems from the need to accommodate varied data characteristics, ensure error correction, and maintain data retrieval efficiency, which often leads to trade-offs in storage density. Therefore, more specialized approaches are necessary to unlock the full potential of DNA storage for medical data.

By tailoring coding algorithms to specific types of medical data, we can optimize the coding process to better suit the characteristics of each data type, thereby maximizing data density. For instance, CT scans, which occupy significant storage space, can be directly converted into DNA sequences using algorithms that fit their unique patterns. In addition to conventional encoding strategies, hierarchical encoding schemes can further compress high-resolution medical images by encoding redundant pixel information more efficiently, potentially enhancing DNA storage efficiency by orders of magnitude. Recent advances in deep learning offer another transformative opportunities for medical data encoding. Deep learning frameworks, such as CNNs, RNNs, and autoencoder architectures, enable adaptive compression of complex data by extracting intrinsic features and learning hierarchical representations. These models have demonstrated remarkable success in image and text compression by identifying patterns that traditional coding methods might overlook. For instance, CNNs can compress pixel-rich medical images like X-rays by identifying common spatial features, while autoencoders can reduce genomic data by learning compressed latent representations. When integrated with DNA storage systems, such models not only improve storage density but also enhance data retrieval efficiency. Furthermore, the prevalent use of templates in hospitals for recording medical data, due to their significant similarity, suggests a promising avenue for enhancing DNA storage coding efficiency. Separating medical data into templates and content, and using a single template for various pieces of content, theoretically offers a substantial opportunity to increase coding density. For instance, patient records often follow standardized formats. By developing coding strategies that recognize and utilize these standard templates, we can separate the static template from the dynamic content, coding each separately. This not only enhances coding efficiency but also simplifies updates and modifications, as changes can be made to the content without re-coding the template. Certain strategies involve building different data blocks representing templates and content, such as constructing short sequences of data blocks and splicing them into longer sequences [82], and segmenting long sequences into multiple overlapping small segments [83].

By leveraging the repetitive and structured nature of medical data, utilizing advanced segmentation and template-based coding strategies, prioritizing hybrid neural network models, and directly converting medical data into DNA sequences, we can significantly enhance the coding density and efficiency of DNA storage systems. However, to achieve practical applications, further research is essential to develop standardized protocols and ensure that neural network models can be seamlessly integrated into existing DNA storage workflows. This will require advancements in bioinformatics and machine learning interfaces specifically tailored for medical data encoding.

### 3.4 Synthesis and sequencing of DNA

The synthesis and sequencing of DNA, driven by commercial market and research demand, significantly contribute to DNA storage by serving as a data writer and reader. Yet, these processes are also the main sources of the high costs and slow speeds that currently limit DNA storage. Moreover, many of the errors encountered in the DNA data storage channel can be traced back to the DNA synthesis and sequencing processes [84].

#### 3.4.1 DNA synthesis

The DNA synthesis market is poised for significant expansion, forecasted to grow from 3.2 billion USD in 2023 to 10.5 billion USD by 2030 [85], fueled by advancements in synthetic biology, genomics, next-generation sequencing (NGS), and personalized medicine. Last year, a global gene synthesis market report stated that the current cost of gene synthesis is approximately 0.09 USD per base pair [86]. Although the cost of DNA synthesis for data storage is higher, it is expected to drop to around 0.01 USD per base pair in the future. Despite this anticipated reduction, these costs are still considerably higher than those of the traditional silicon-based data writing processes.

The predominant approach for DNA synthesis is the chemical synthesis, especially the solid-phase phosphoramidite triester method, favored for its well-established technology and cost-effectiveness. Yet, it faces significant challenges, including incomplete chemical reactions and side reactions, which elevate the error rate in DNA synthesis and decrease the yield, especially as oligonucleotide chain lengths grow. For instance, at a 99 % elongation cycle efficiency, the theoretical yield drops from about 30 % for a 120-nucleotide oligonucleotide (0.99^120^ × 100 %) to merely 13 % for a 200-nucleotide sequence (0.99^200^ × 100 %). Consequently, the feasible maximum length for synthetic nucleotide chains hovers around 300 nucleotides. Moreover, the extensive use of toxic chemicals in the synthesis process necessitates rigorous waste disposal measures for both liquid and gaseous by-products. In response, various techniques are being developed to improve efficiency and yield, extend oligonucleotide length, and mitigate environmental impact.

Enzymatic DNA synthesis, particularly the terminal deoxynucleotidyl transferase (TdT) enzyme-mediated synthesis, marks a significant shift in the field. TdT, being template-independent, allows for efficient polynucleotide chain elongation in an aqueous environment, sidestepping pollution concerns and offering the promise of a more cost-effective, longer DNA molecule synthesis. Lee et al. demonstrated the potential of this method by coding digital data into DNA strands with short homopolymeric extensions, leveraging the transition between non-identical nucleotides [87]. Further advancements could see engineered TdT variants achieving controlled nucleotide incorporation, enabling the synthesis of precise, user-defined DNA sequences [[88], [89], [90]]. Reflecting this potential, a 2020 report highlighted numerous start-ups pursuing enzymatic synthesis as a pathway to quicker and more efficient synthesis of long DNA strands [91].

Ligation synthesis represents another innovative approach. The BGI Institute of Life Sciences in Shenzhen, China provided a proof-of-concept of DNA ligation synthesis and completed a patent application in text storage [92]. The basic principle is mapping Chinese characters to pre-synthesized double-stranded DNA (dsDNA) blocks, each equipped with a base end for attachment to specially designed adapters. This method allows for the assembly of blocks into desired sequences through adapter homology. Using a similar method, the US company Catalog successfully assembled 16 GB of data from Wikipedia using pre-synthesized DNA sequences in 2019, although no technical details were disclosed [93]. Later in 2021, they announced the development of a custom DNA writer capable of encoding data at 1 Mbps into DNA through combinatorial assembly [94]. In 2023, Liu's team made an application of ligation synthesis in DNA storage using the ancient movable type printing principle [95]. They created modular DNA blocks by synthesizing and immobilizing data-encoded sequences in DNA hairpins onto solid beads. These blocks were then replicated and sequentially attached to primers using a DNA polymerase-catalyzed primer exchange reaction, allowing for the recording of new information. This year, Leblanc et al. proposed a fully *in vitro* protocol to generate very long dsDNA molecules starting from commercially available short DNA blocks using Golden Gate assembly [96]. They successfully streamlined the process and produced a 24 KB-long DNA molecule storing part of the Declaration of the Rights of Man and of the Citizen of 1789.

Some groups made further improvements by combining enzymatic synthesis and ligation synthesis. Yan et al. used a Bridge Oligonucleotide Assembly (BOA) technology to assemble enzymatically-ligated composite motifs to reduce synthesis costs and increase storage density [97]. Simmons et al. assembled enzymatically synthesized short pieces of DNA into longer sequences [98]. The results indicate that DNA fragments synthesized by the enzymatic method show high accuracy and reliability in gene assembly, which is of great significance for the fields of synthetic biology and genetic engineering.

#### 3.4.2 DNA sequencing

According to a DNA sequencing market size and share analysis report by Mordor Intelligence, the size of the DNA sequencing market was approximately 11 billion USD in 2022 and is expected to grow to 24 billion USD by 2028 [85]. The major drivers for the market growth include advancements in DNA sequencing technology, increasing applications in clinical diagnostics and drug discovery, and growing investment in research and development. The reduction in DNA sequencing costs has exceeded that of DNA synthesis and outpaced Moore's Law by a significant margin. The price of DNA sequencing per human genome has been dropping from 10 million USD in 2006 to around 1000 USD in 2015, and continues to drop. In recent years, the price began leveling off and has been remaining around 500 to 600 USD per human genome [99]. But in May 2022, a news report claimed that Ultima Genomics (Newark, CA, USA), a heavily funded start-up with 600 million USD in venture capital, has developed a novel sequencing technology that achieves the long-sought goal of 100 USD per human genome [100]. However, no specific detail has been demonstrated until now.

Due to its high throughput, low cost, fast speed, and wide range of application, the most dominant sequencing technology is the NGS, led by companies like Illumina. However, the read length limit of NGS impedes its application in DNA storage. To overcome this limitation, third-generation sequencing (TGS) was introduced. In 2011, Pacific Biosciences (PacBio) introduced a novel third-generation sequencing (TGS) technology called "single-molecule real-time (SMRT)" sequencing, which allows for the sequencing of long DNA fragments [101]. According to PacBio's official information, SMRT sequencing can achieve read lengths of tens of kilobases [102]. More recently, in 2014, Oxford Nanopore Technologies (ONT) introduced a nanopore sequencing technology termed "MinION", which uses a nanopore to determine the DNA sequence by measuring changes in electrical current as single-stranded DNA molecules pass through the nanopore [103]. Nanopore sequencing offers not only long read lengths but also portability and real-time analysis capability. Research has demonstrated that MinION sequencing can identify the DNA of target bacteria using a portable device within 30 min [104]. Although promising for DNA storage, TGS still lags behind NGS in terms of cost, throughput, and accuracy.

Nowadays, the reliance on established commercial DNA chemical synthesis and NGS technologies is pivotal due to their proven reliability, efficiency, and scalability in handling the vast amounts of data generated in healthcare. DNA chemical synthesis methods, particularly solid-phase phosphoramidite synthesis, have demonstrated high accuracy and scalability for encoding medical cold data. NGS remains the gold standard for reading DNA-encoded data due to its high throughput and affordability. These technologies have formed the backbone of current DNA storage systems by providing a balance between performance, cost, and compatibility with healthcare data management requirements.

Although novel developments in DNA synthesis and sequencing present prospective and promising applications, they are currently impractical for the DNA storage workflow. For instance, enzymatic DNA synthesis methods have emerged as a greener and potentially more scalable alternative to chemical synthesis. However, challenges related to nucleotide incorporation accuracy and controlled synthesis length have yet to be resolved for practical applications. Similarly, TGS technologies such as SMRT and nanopore sequencing offer long-read capabilities and portability, but their current limitations in sequencing accuracy and cost make them less ideal for the high-fidelity requirements of medical cold data.

In the near future, well-established commercial DNA chemical synthesis and NGS technologies will continue to be foundational for medical cold data DNA storage systems. To enhance their applicability, incremental improvements in error correction algorithms and synthesis optimization are necessary. Strategies such as enzymatic error correction, primer redesign for PCR-based sequencing, and automated synthesis platforms can help reduce the error rates and costs associated with current workflows. Additionally, hybrid approaches that combine chemical and enzymatic synthesis may offer a promising pathway for balancing synthesis accuracy and environmental sustainability. As the medical market expands and technological innovations drive further cost reductions and performance improvements of DNA synthesis and sequencing, they will enable the healthcare industry to harness the full potential of DNA storage.

### 3.5 Random access

DNA is primarily synthesized and sequenced *de novo*, posing significant challenges for the random writing and reading capabilities in DNA storage. Although random writing is less crucial for storing medical cold data, a certain level of random access capability is still required to manage the substantial data volume.

Traditional DNA data access has relied on the well-established and cost-effective PCR technology. Back in 2008, Yamamoto et al. implemented a DNA memory with more than 10 million (16.8 MB) addresses [105]. They used nested PCR to selectively amplify specific sequences in DNA solution to extract the target sequence of a specific address. However, several drawbacks have hindered the application of PCR in DNA storage. First, the PCR process is time-consuming, requiring a few hours to days. Secondly, in traditional PCR, a separate forward and reverse primer must be designed for each DNA segment to be amplified. This results in high redundancy, as multiple unique primers are needed for different fragments, and lowers the overall efficiency of the process.Lastly, using PCR for random access amplifies both the target and background files, reducing efficiency and increasing time consumption.

To address these issues, innovative approaches have been developed. In 2018, Organick et al. developed a method using unique primers for unique DNA sequences, thereby reducing primer numbers and enhancing efficiency [20]. They encoded and stored 35 distinct files, totaling 200 MB, in more than 13 million DNA oligonucleotides (about 2 billion nucleotides in total) and fully recovered the data with no bit errors, not only improving simplicity and efficiency but also reaching an ideal target sequence extraction rate. Later in 2019, Tomek et al. introduced a label-based magnetic sorting method, integrating chemical handles with magnetic beads to selectively bind DNA sequences [106]. The team designed 10 primers to uniquely identify and retrieve 72 files, achieving a 97 % retrieval rate from 5 Terabyte (TB) of data, albeit with more complex protocols and costlier reagents. In 2022, Claris Winston implemented efficient and selective oligo retrieval using a combinatorial PCR primer approach, using 9 forward and 9 reverse primers to specify 81 unique files [107]. Although the efficiency is slightly lower than before, it only employed a simple standard PCR procedure with less cost and higher efficiency to reach an extraction rate of more than 99 %. To further explore the characteristics of PCR, Tomek et al. developed a file preview operation in DNA-based data storage using different nonspecific amplification levels in different environments [108]. Theoretically, only the exact primer and DNA pairs would bind together. Still, Tomek's team discovered that lower annealing temperatures and higher primer concentrations will increase nonspecific amplifications, and vice versa. Therefore, the authors divide a file into multiple similar sequences and design a homologous primer for them. The primer will combine with less sequence to provide a preview in stringent environments, and combine with all the sequences to provide high-definition images in promiscuous environments. Employing this method, they successfully previewed four image files against a randomized, nonspecific 1.5 GB background in a DNA storage system.

In addition to PCR, various approaches have been explored for random access in DNA storage. Lau et al. introduced a DNA storage system employing magnetic separation technology, termed "Magnetic DNA-based Random Access Memory (MDRAM)", which achieved repeated and efficient object file reading through the combination of synthetic DNA and magnetic agarose beads [109]. Banal et al. encapsulated data-encoded DNA into silica particles, addressing content barcodes with orthogonal 25-nucleotide ssDNA strands. Fluorescently labeled 15-nucleotide ssDNA probes complementary to the file barcodes were used to retract files using fluorescence-activated sorting (FAS) [110]. This physical separation and access of DNA can obviate the need for numerous heating and cooling cycles and enzymatic synthesis, thereby eliminating non-specific crosstalk between file sequences and barcodes in the existing PCR-based file systems. Using a similar encapsulation approach, Bögels et al. stored DNA in thermoresponsive microcapsules and implemented a fluorescence-based DNA file retrieval by scanning flow cytometry (FACS) [111]. The membrane of thermoresponsive microcapsules becomes penetrable at low temperatures, allowing the passage of enzymes, primers, and amplification products, and contracts at high temperatures to prevent molecular crosstalk during amplification. Applying machine learning methods, Bee et al. used the similarity of DNA sequences to conduct similarity searches in the DNA database of 1.6 million images [112]. The image features were converted into DNA sequences, and similar DNA sequences were identified using hybridization probes to search common image features. This molecular-level similarity search in DNA databases achieves high efficiency and accuracy, comparable to the performance of existing electronic algorithms. From a coding aspect, El-Shaikh et al. introduced a coding method for executing content-based filtering queries on a DNA data storage system [113]. The method makes use of the content-based barcodes (CBBs) to encode information into DNA sequences that also act as DNA strand tags and enables the DNA storage system to search directly based on the content, without relying on other mapping information. This approach reduces sequences that do not code actual information, thereby improving the efficiency and flexibility of data access. Simalarly, Su et al. introduced a content-based coding method that utilizes non-specific hybridization of DNA sequences for instance-based learning [114]. This approach encodes handwritten digit images from the MNIST dataset into DNA sequences, allowing for direct classification based on the content of the sequences with an average accuracy of 95 %.

*In vivo* random access is less developed due to the complexities of cell manipulation. However, certain explorations involved the high-throughput microfluidic single-cell digital PCR technology, allowing parallel processing of individual cells for cell capture, washing, lysis, reverse transcription, and digital PCR analysis [115]. Although it is designed for the detection of mRNA transcripts and micro-RNAs, we believe that certain adjustments will improve the feasibility for DNA. Leveraging gene editing tolls, Zhang et al. present Search Enabled by Enzymatic Keyword Recognition (SEEKER), which utilizes CRISPR/Cas12a to rapidly generate visible fluorescence when a DNA target corresponding to the keyword of interest is present [116]. SEEKER achieves quantitative text searching because the growth rate of fluorescence intensity is proportional to keyword frequency. Regarding random data access of *in vivo* DNA storage, a recent research storing data in motion-restricted *Escherichia coli* and arranging bacteria through spatial distribution, showing promise for achieving micro-scale random data access [117].

For medical cold data random access, accuracy and cost-effectiveness are crucial, while time consumption is more tolerable. Unlike hot data systems that require immediate and frequent access, cold data in the healthcare context often involves archival information that is accessed infrequently but must remain intact and accurate over extended periods. Therefore, reliability and data integrity take precedence over rapid access.

In the near term, enhancements to traditional PCR methods present a pragmatic solution that balances these priorities effectively. Traditional PCR technology, which is inexpensive and well-established in molecular biology, provides a robust framework for selectively amplifying specific DNA sequences from a complex mixture. PCR is particularly suitable for medical cold data storage due to its ability to target and amplify precise DNA fragments without requiring the entire dataset to be accessed, reducing processing complexity and resource consumption. By refining PCR techniques, it is possible to enhance their efficiency and specificity, making them more suitable for the demands of large-scale DNA data storage. Recent innovations such as combinatorial PCR, which reduces primer redundancy, and nested PCR, which improves specificity, have demonstrated their potential for optimizing DNA data retrieval processes. Additionally, primer redesign strategies can reduce cross-contamination between target sequences, enhancing retrieval accuracy even in complex DNA pools. While the time required for PCR amplification is longer compared to electronic data retrieval, this is not a critical issue for medical cold data, which is accessed infrequently. Moreover, advancements in high-throughput PCR platforms have significantly shortened amplification times, making them more viable for large-scale data retrieval. These platforms can process multiple samples simultaneously, thereby increasing throughput and efficiency.

By leveraging the established strengths of PCR technology and incorporating recent advancements, we can develop robust, cost-effective, and reliable DNA-based random access systems for medical cold data storage. However, further research is necessary to explore alternative approaches such as magnetic bead-based retrieval systems and microfluidic-assisted PCR platforms, which offer additional scalability and automation for future DNA storage systems, which will be critical to ensuring high-fidelity data retrieval.

### 3.6 More than A, T, C, G

Traditionally, DNA storage has relied on the arrangement of the four bases to record and store information. This method leverages the natural structure of the DNA sequence and takes advantage of its stability and programmability. However, this method also suffers from certain inherent disadvantages. To extend the 4-base algorithm, researchers have constructed artificial nucleotides to add more coding units and increase coding density. Meanwhile, researchers have been utilizing the self-assembly properties of DNA to design nanostructures for data storage. The different structures and forms of DNA nanostructure can serve as coding units, similar to bases in DNA.

#### 3.6.1 Artificial nucleotides

The structure of natural DNA comprises deoxynucleotides with different bases, including A, G, C, and T. Artificial nucleotides have been created by partially replacing and/or chemically modifying natural DNA components. One of the first reports involved an enzymatically formed base pair of iso-cytosine and iso-guanine in *Escherichia coli*, resembling the natural A:T and C:G base pairs [118]. In 2020, Yang et al. developed α-l-threofuranosyl nucleic acid (TNA) by replacing DNA's natural five-carbon deoxyribose sugar with an artificial four-carbon threose sugar, enhancing resistibility against biological nucleases [119]. In recent years, through the use of chemically modified nucleotides [120], degenerate bases [121] and 2′deoxyisonucleotide triphosphate based iso-nucleotides [122], the DNA storage alphabet has been expanded to 11, 12, and 15 letters, respectively, significantly improving coding density. In addition to these advancements, recent research has also explored epigenetic modifications, such as DNA methylation, for data encoding [123]. Unlike expanding the DNA storage alphabet, this method takes advantage of the controllability and reversibility of chemical modifications to directly encode binary data into DNA. This approach simplifies the encoding process, making it more efficient and reducing the reliance on the specialized synthesis of DNA strands. As a result, it significantly lowers storage costs while accelerating the data storage process, providing a faster and more convenient solution for DNA-based data storage.

#### 3.6.2 DNA nanostructures

Among all natural or synthetic molecules, DNA is the most predictable and programmable one [124]. Naturally, DNA has the ability to self-assemble into a double-stranded double-helix structure. Using this ability, DNA strands can be hybridized into specific nanostructures, whose arrangement and alteration can record and store information. Different assembled structures can be identified using atomic force microscopy (AFM) or nanopore sequencing. DNA nanostructures are characterized by high-speed read and write capabilities, ease of erasure and rewriting, and robust encryption [125]. Although larger than individual nucleotide bases, DNA nanostructures can still offer several orders of magnitude improvement in data density compared to silicon-based storage approaches.

In DNA nanostructure-based data storage, representing binary digits "0" and "1" involves the creation of unique patterns. Early demonstrations in 2004 involved a system with 8 memory states using 3 single-stranded overhangs in DNA [126]. The overhangs, structured scaffolds formed by assembling partially complementary DNA strands, were designated as "0" and could be converted to "1" by changing to double-stranded duplexes through the introduction of complementary strands. Later in 2006, Rothemund et al. developed the concept of DNA origami by folding single DNA strands onto themselves following desired patterns, thereby transforming the landscape of DNA nanotechnology [127]. Using the DNA origami method, Zhang et al. assembled biotin-modified DNA into specific patterns to store text, musical notes, and images, which could be securely read out using AFM [56]. Dickinson et al. on the other hand, wrote data on DNA origami using the present state of ssDNA with docking-site domains and then read it out by monitoring the binding of fluorescent image probes using DNA-PAINT [128]. Another recording method using DNA nanostructure is leveraging the DNA hairpins. The Keyser team successively used the present state of DNA dumbbell hairpins [129] and the states of 8 bp and 16 bp hairpins [130] as representations of the bit states for data storage. Assembling DNA on carrier materials can also serve as data storage devices, such as forming unique DNA nanostructure patterns on wafers [131] and gold surfaces [132], condensing DNA strands on the surface of carbon nanotubes [133], and attaching DNA multi-way junctions of different sizes to a double strand of DNA carriers [134]. The modification of dsDNA topology has also been harnessed for data storage. Tabatabaei et al. achieved successful data storage by introducing nicking positions in dsDNA [135]. Chandrasekaran et al. used site-specific modification of DNA nanostructures to store and rewrite information, designing addressable structures that allow write, erase, and rewrite operations at specific locations [136].

Artificial nucleotides expand the genetic alphabet beyond the four natural bases, potentially increasing coding density and data storage capabilities. By introducing novel base pairs, artificial nucleotides can theoretically improve information density and enhance data retrieval fidelity, particularly for complex and structured data types such as genomic sequences. Recent advancements in chemical synthesis have enabled the creation of synthetic bases with unique bonding properties, which may support next-generation DNA storage systems. DNA nanostructures leverage the self-assembly properties of DNA to create complex structures that can code information in novel ways. These nanostructures, such as DNA origami and multi-helix bundles, offer potential for high-density storage through spatial encoding. They can also provide robust encryption mechanisms by forming complex, difficult-to-predict patterns that add an additional layer of security for sensitive medical data.

Although artificial nucleotides and DNA nanostructures provide solutions to specific drawbacks of traditional DNA storage, they are currently far from practical applications. The synthesis and manipulation of artificial nucleotides and DNA nanostructures require highly specialized laboratory conditions and expertise, which are not yet feasible for widespread commercial deployment. Moreover, maintaining the chemical stability and structural integrity of these synthetic components remains challenging, especially under the environmental conditions required for long-term medical cold data storage. The processes involved are often complex, costly, and time-consuming, lacking the scalability needed for integration into routine medical cold data storage systems. In addition, compatibility with existing DNA synthesis and sequencing technologies is limited, requiring the development of new protocols and instrumentation for efficient data writing, reading, and accessing. These factors currently hinder the adoption of artificial nucleotides and nanostructures for large-scale medical cold data storage. Furthermore, clinical genomic data is expected to be significant and massive in the future healthcare field. Rather than converting them into certain substitutions, storing them in their original form is more convenient and cost-effective. Therefore, the traditional four-base DNA storage model remains the most suitable and reliable choice for medical cold data storage in the near term. Its established synthesis, storage, and sequencing protocols offer a stable, scalable, and cost-effective solution. However, continued research into artificial nucleotides and DNA nanostructures should not be overlooked, as advancements in these areas may provide innovative solutions for specialized storage applications in the long term, particularly where high data density or advanced encryption is required.

### 3.7 Automation and integration

Although DNA storage technology surpasses modern storage devices in terms of storage density, durability, and energy consumption, it faces a significant gap in data reading and writing speeds. The cost and speed gaps for reading are 10^8^ and 10^5^ times greater, respectively, while those for writing are 10^5^ and 10^2^ times greater [137]. Moreover, most of the existing commercial synthesis and sequencing systems involve cumbersome equipment requiring a large workplace and specialized operation, rendering it an impractical choice for hospitals. A recent article argued that automated DNA synthesis and sequencing are the keys to unlocking virtually unlimited data storage capacity [138]. Therefore, automating and integrating DNA storage systems is crucial for their practical application.

Given the liquid-state nature of DNA manipulation, microfluidic technology for the manipulation of tiny droplets has become highly suitable for the DNA storage workflow. It offers advantages such as high degrees of miniaturization, efficiency, speed, integration, and automation. Microfluidic platforms have been developed for various aspects of DNA storage.

#### 3.7.1 DNA synthesis using microfluidics

Several microfluidic platforms have been developed to improve the efficiency and reduce reagent consumption of DNA synthesis. In 2004, a study introduced the microfluidic PicoArray technique, which synthesizes oligonucleotides and simultaneously assembles multiple DNA sequences efficiently [139]. Later in 2007, Huang et al. presented a solvent-resistant microfluidic DNA synthesizer capable of synthesizing 20 nucleotide oligonucleotides, highlighting its efficacy in reducing reagent consumption and producing high-quality DNA oligonucleotides [140]. In 2010, Lee et al. designed a microfluidic oligonucleotide synthesizer that efficiently synthesizes oligonucleotides in minimal reaction volumes, thus significantly reducing reagent consumption [141]. Recent developments have aimed to enhance specific capabilities of DNA synthesis. Antkowiak and coworkers reported a low-cost DNA data writing device with a rapid light-directed array platform [142]. Data amounting to 1.3 MB were synthesized and successfully recovered on the platform at a low cost of 530 USD per MB. Utilizing a similar light-triggered approach, Lee et al. conducted a multiplexed enzymatic DNA synthesis of 12 unique data-encoded DNA sequences (110 nucleotides) through the rapid uncaging of Co2+ ions by patterned UV light to activate TdT in a microfluidic flow cell [87]. The proposed photon-directed multiplexed enzymatic DNA synthesis may become a cleaner, faster, and more flexible synthesis methodology for DNA storage. To increase density, researchers at the University of Washington developed the first nanoscale DNA storage writer, employing electrochemical array technology to cluster millions of nanoelectrode holes in an area under 1 square micron [143]. They successfully realized the writing, reading, and decoding of 40-byte messages. The writing density of DNA reached 25 × 10^6^ sequences/cm^2^, which is an improvement of three orders of magnitude over the existing DNA synthesis arrays. And the quality of synthesized DNA has been proven sufficient for DNA data storage. Focusing on the error rate, Khilko et al. optimized Gibson assembly, PCR, and enzymatic error correction reactions on a droplet digital microfluidics (DMF) platform [144]. With this method, a dsDNA sequence of 339 base pair (bp) was successfully assembled from 12 oligonucleotides, and the error frequency was reduced to 1.8 errors/KB after a round of error correction. To increase the length of the nucleotide, Yu et al. presented a droplet-based fluidics system for DNA ligation synthesis. This method involves synthesizing pieces of DNA of a specific size and assembling them programmatically in a droplet [145].

#### 3.7.2 DNA retrieval using microfluidics

The first notable approach regarding DNA retrieval employs a DMF platform, which controls individual droplets to retrieve dry DNA powders organized in an array format [146]. DNA powder is stored on a separate glass slide, with the DMF platform being used to redissolve the powder and direct it to precise locations for further processing. Electrowetting facilitates the manipulation of individual droplets, whose movement is effortlessly controlled via voltage adjustments and computer programming. This method has demonstrated efficient retrieval of substantial DNA quantities and successful file recovery from specific storage spots on the glass slide, with minimal cross-sample contamination. Another established microfluidic platform, characterized by a two-layer polydimethylsiloxane (PDMS) micro-valve network architecture, enables a comparable degree of automated manipulation of liquid samples [147]. Recently, a microfluidic platform has been designed for the automated storage and retrieval of data-encoded oligonucleotide samples via a network of microvalves [148]. The study demonstrated a capability of achieving up to 150 TB of storage equivalence in a 3 × 7 cm^2^ device footprint, marking a significant advancement in system integration, device miniaturization, and process automation for DNA storage. Utilizing electrically controllable gold electrodes, Athreya et al. have demonstrated nanofluidic devices capable of capturing, retaining, and subsequently releasing chimeric DNA molecules [149], potentially further miniaturizing DNA synthesis platforms.

#### 3.7.3 DNA sequencing using microfluidics

Integrating microfluidics into DNA sequencing seeks to surmount the challenges posed by bulky instruments and intricate procedures. A recent breakthrough is the MinION, a compact four-inch USB device. This innovation facilitates real-time data reading in DNA storage systems to deciphers information stored in DNA strands through a DNA assembly strategy, proving to be faster and more flexible than traditional sequencing methods. In 2017, the first portable system for DNA sequencing utilizing MinION nanopore sequencers was introduced [24]. Subsequently, in 2019, Lopez et al. successfully demonstrated a complete data storage process—amplifying, assembling, and reading three files amounting to 1.67 MB of digital information stored in 111,499 oligonucleotides—leveraging nanopore sequencing for its long-read capabilities [150]. MinION offers benefits including portability, cost-effectiveness, and ease of integration, making it a good chice to integrate with microfluidics for the improvement of DNA sequencing. The first example to combine MinION and microfluidics was achieved by Liu et al., in 2021 [104]. They combines digital microfluidics (DMF) and MinION sequencing by automating bacterial DNA amplification through DMF's precise droplet control, followed by MinION's real-time nanopore sequencing. This integrated system achieves rapid (3-h) pathogen detection with 100x higher sensitivity than conventional methods, while maintaining portability and minimal manual operation for point-of-care diagnostics. From a more integrating perspective, Liu's team developed the first integrated DNA data storage system enabling both DNA synthesis (writing) and sequencing (reading) on a single microfluidic chip [151]. The system employs a SlipChip-based microfluidic platform where sliding operations precisely align reagent droplets with a gold electrode array for electrochemical synthesis. This design simplified liquid control, reduces reagent consumption (<5 % waste), and achieves full workflow integration without device disassembly.

#### 3.7.4 Sample preservation and manipulation using microfluidics

Microfluidics can also be integrated into the preservation and manipulation of DNA samples. In 2022, Antkowiak et al. integrated DNA encapsulation with digital microfluidics for automated DNA data storage, verifying the stability and compatibility of packaged DNA with DMF systems [152]. To advance microfluidics in DNA sample manipulation, a workflow encompassing DNA extraction, fragmentation, adapter ligation, and amplification was developed in 2019, offering an efficient, integrated microfluidic platform for sample preparation prior to NGS [153]. In 2023, Geng et al. showcased an automated and non-destructive technique for extracting encapsulated DNA via a nanoparticle-coated microfluidic chip [154]. Considering both encapsulation and extraction of DNA, Mao introduced a novel method merging metal-organic frameworks (MOFs) with microfluidic technology for the automated and integrated storage of DNA data [155]. The method uses a microfluidic chip to complete DNA encapsulation in 10 min and extraction in 5 min, significantly improving the stability of DNA and realizing the automation and integration of data storage. Regarding the *in vivo* approach, microfluidic devices facilitating cell manipulation—encompassing capture, purification, lysis, reverse transcription, and digital PCR analysis—could advance the development of *in vivo* DNA storage [156]. Recently, Iwai et al. developed a droplet microfluidic chip for executing up to 100 independent CRISPR gene editing reactions. They used the chip to demonstrate the disruption of the galK gene in *E. coli* and the engineering of improved indigo pigment synthesis strains [157].

Microfluidic technology holds great promise for automating and integrating DNA storage workflows. By manipulating tiny droplets in micro-scale environments, microfluidics can precisely control DNA synthesis, sequencing, and preservation processes. This miniaturization reduces reaction volumes, optimizes reagent usage, and enables highly parallelized operations, making it a promising avenue for future advancements in DNA storage. These advantages are particularly appealing for handling medical cold data, where long-term stability and high data integrity are crucial.

A research team has developed a fully automated DNA data storage device, integrating modules for coding/decoding, DNA synthesis, and DNA preparation and sequencing [158]. The experiment verified the system's validity by successfully storing and retrieving the 5-byte message "HELLO" within 21 h. This demonstration highlights the feasibility of integrating multiple DNA storage operations onto a single microfluidic platform, paving the way for future automated solutions. Similarly, another team in Brazil conducted an experiment involving DNA storage, including data coding, decoding, and retrieval using microfluidic technology [159]. Despite its immense potential to revolutionize DNA data storage, the development of reliable and robust microfluidic systems capable of meeting the specific demands of large-scale medical data storage remains in its early stages and is unlikely to materialize in the near future. Current challenges include the need for improved material compatibility, error-prone microfluidic operations, and the complexity of scaling up production for high-throughput applications. Given this situation, the mature DNA synthesis and sequencing platforms currently in use will continue to be the mainstay for medical data DNA storage in the near term. These platforms have demonstrated high reliability, throughput, and compatibility with existing medical data workflows, which are essential for immediate deployment in healthcare settings. For the foreseeable future, the DNA storage system of medical cold data will rely on larger and complex machines and platforms. These systems, although not as compact or integrated as microfluidic devices, offer the necessary capacity, reliability, and cost-effectiveness for handling vast amounts of medical data. However, as microfluidic technology matures, its integration into DNA storage systems has the potential to streamline processes, reduce operational costs, and enable more flexible and scalable storage solutions. Collaborative efforts between microfluidics engineers and DNA storage researchers will be essential to overcoming current limitations and unlocking the full potential of this technology.

### 3.8 Establishment of DNA storage centers

**Fig. 3:**
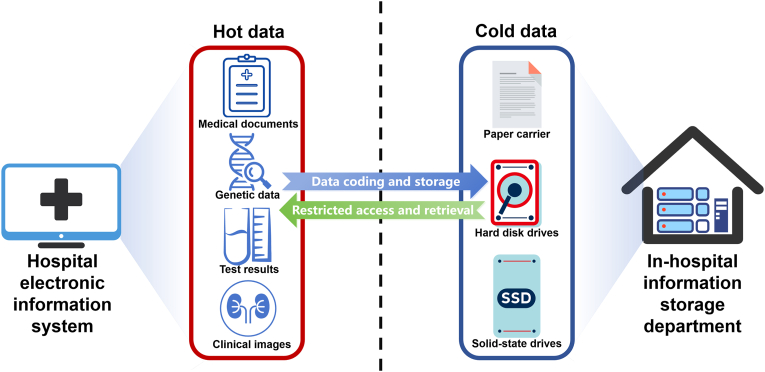
**Illustration of Traditional Medical Data Storage in Hospitals.** Medical data is first converted into binary format within the hospital's electronic information systems as hot data to facilitate daily access and read/write operations. After diagnosis and treatment procedures, the data is categorized as cold data for long-term storage, typically saved on paper carriers, hard disk drives, or solid-state drives, and preserved in the hospital's information storage department. The stored data can be accessed and retrieved under specific requests, with access restrictions in place to ensure data security and confidentiality.

Currently, hospitals typically manage and store medical data on-site (Fig. 3). In a hospital setting, medical data is first converted into binary format within the hospital's electronic information systems as hot data to facilitate daily access and read/write operations. After diagnosis and treatment procedures, the medical data is categorized as cold data for long-term storage, usually saved on storage media such as silicon-based devices or paper carriers, and preserved in the hospital's information storage department. When specific data is required, hospital personnel submit a request to the information storage department to access the relevant storage media and retrieve the original data. While this method ensures the integrity and security of medical information, relying on traditional storage media also poses challenges in terms of energy consumption, space utilization, and longevity.

One possible solution is DNA storage. However, establishing dedicated DNA storage departments in hospitals may present significant challenges, including equipment, physical space, and personnel requirements, potentially exceeding affordability limits given the current state-of-the-art technology. Moreover, since a significant portion of medical data falls into the category of cold data, characterized by infrequent reading, writing, editing, and maintenance operations, establishing DNA storage departments might lead to low cost-performance ratios. Additionally, DNA chemical synthesis, likely the dominant method for data writing in DNA storage, presents challenges. The generation of chemical waste and harmful substances from DNA chemical synthesis poses risks, especially in hospital environments with high human activity.

To address the challenges of hospital-centric DNA storage, we propose to establish regional DNA storage centers (RDSCs). RDSCs would centrally store medical cold data from multiple hospitals within a region, enabling a more streamlined and secure management system. The initial step in the RDSC model involves data transmission. To connect multiple hospitals to one RDSC, internet data transfer is the optimal way. Connectivity between hospital information systems and RDSCs would be secured through encrypted transmission protocols to safeguard data privacy and security. Hospitals would transmit their data to RDSCs via secure, encrypted channels, ensuring that all data remains protected from unauthorized access during transit. The use of VPNs (Virtual Private Networks) and SSL (Secure Sockets Layer) encryption could be considered to enhance security measures. Then, data arriving at RDSCs would undergo format standardization. Medical data from different hospital systems would likely be presented in different formats. Therefore, a uniform data protocol would be established, converting data from various hospitals into a standardized format compatible with DNA storage technology. This process involves using sophisticated software tools that can automatically convert medical information into a structured format suitable for coding into DNA sequences efficiently. By ensuring that all incoming data adheres to this standardized format, RDSCs can streamline the coding process and maintain data integrity. After that, a formal DNA storage workflow would be conducted in RDSCs. Facilities specialized in molecular biology, genomics, and data management would be set up for the management of the DNA storage workflow. The staff would oversee the coding of digital data into DNA, the storage of DNA in bio-stable conditions, and the decoding of DNA back into digital format when data retrieval is requested. As the anticipated manpower consumption for RDSC daily maintenance is limited, we believe that it would be cost-effective. To avoid pollution caused by DNA chemical synthesis and reduce site costs, the site selection for RDSCs would be better in sparsely populated areas. However, factors such as proximity to cluster hospitals, accessibility, and internet stability also need to be considered. Within the facilities, not only for DNA samples, but also for the instruments, controlled temperatures and humidity levels are necessary to ensure long-term stability. Also, cleanroom environments would be prioritized to prevent contamination and ensure the integrity of the DNA samples.

The concept of RDSCs represents a transformative approach to managing the ever-increasing volumes of medical cold data, offering a comprehensive and forward-thinking solution. These centralized facilities are designed to handle large-scale data storage needs by aggregating resources from multiple hospitals within a region. By utilizing internet data transfer, centralizing data storage, optimizing resource utilization, mitigating environmental concerns related to DNA chemical synthesis, and leveraging the unique properties of DNA, RDSCs would provide a secure, efficient, cost-effective, and sustainable alternative to traditional hospital-centric storage methods.

## 4 Designing a DNA storage system for medical cold data

**Fig. 4:**
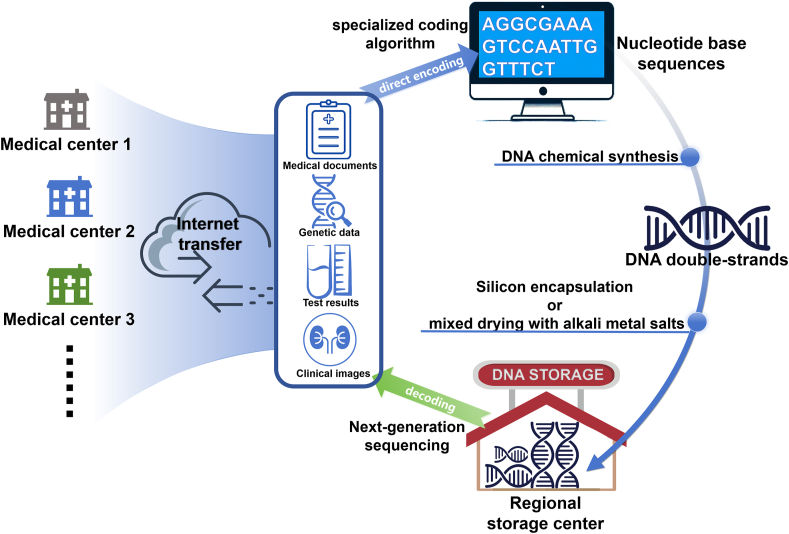
**A DNA storage system for medical cold data.** Medical data from multiple medical centers are collected using secured internet transfer, and coded into nucleotide bases using medical data specialized coding algorithms. Then, data-embedded nucleotide bases will be synthesized by DNA chemical synthesis methods and yield double-strands DNA. The DNA, along with the medical data, can be encapsulated with silicon or mixed drying with alkali metal salts for long-stem storage in regional storage centers in a sparsely populated area. Data can be retrieved by the NGS and decoded back to original medical data. The whole process is safeguard by traditional digital encryption and DNA steganography.

A prediction suggested that DNA storage technology might become commercially available within 4–10 years [160]. DNA storage, with its numerous advantages over traditional methods, especially for medical cold data storage (Table 2), presents an optimal solution to the growing data crisis in healthcare. In this context, we aim to design a highly efficient DNA storage system specifically tailored to the needs of medical cold data (Fig. 4).

**Table 2:** Comparison between traditional storage and DNA storage for medical cold data.

Aspect	Traditional Storage	DNA Storage	In-depth Analysis
**Overall Cost**	Lower initial investment, but high long-term maintenance costs	High initial cost, but amortized over long-term storage	DNA storage's high initial cost is offset by its long-term durability, making it ideal for cold data that requires extended retention
**Data Longevity**	5–10 years	Over 100 years with proper preservation	DNA storage offers exceptional longevity, perfect for storing medical cold data over many decades.
**Read/Write Costs**	Low-cost read/write operations	High for writing (DNA synthesis); moderate for reading	DNA storage's high write/read costs are manageable for cold data, which requires infrequent access.
**Read/Write Speed**	Milliseconds to seconds	Hours to days	Slow read/write speeds are acceptable for medical cold data, which is rarely accessed.
**Storage Density**	HDDs: ∼1 TB/in^2^ SSDs: ∼2 TB/in^2^	single-stranded DNA (ssDNA): 0.455 ZB/g	DNA's extremely high storage density is ideal for managing the large volumes of medical cold data.
**Energy Efficiency**	Moderate to high energy usage	Extremely low energy consumption	DNA storage requires extremely low energy for retention, making it more energy-efficient for long-term storage of medical cold data.
**Maintenance Costs**	High, due to hardware degradation and replacements	Minimal, with proper preservation	DNA storage needs minimal maintenance, aligning well with the low-maintenance nature of medical cold data.
**Scalability**	Limited by physical hardware constraints	Virtually unlimited scalability	DNA storage offers virtually limitless scalability, ideal for the growing volume of medical cold data.
**Environmental Impact**	electronic -waste, high energy consumption	Lower energy use, but chemical synthesis produces waste	DNA storage is more sustainable, with lower energy consumption and reduced electronic-waste compared to traditional storage.
**Future Adoption**	Established, incremental improvements in density	Expected breakthroughs in cost and speed over decades	DNA storage is a promising future solution for medical cold data due to its unique benefits of longevity, density, and scalability.
**Cost Projection**	Steady decline but reaching physical limits	Expected sharp decline with tech advancements	As DNA storage technology advances, costs will drop, making it more viable for large-scale medical cold data storage.

Given the absence of integrated and automated solutions, along with potential economic and environmental concerns associated with in-hospital DNA storage, the establishment of RDSCs is considered a more pragmatic choice. Storing DNA data within hospitals faces several critical limitations. Economic burdens are one of the most pressing issues, as hospitals are required to invest heavily not only in the initial infrastructure but also in the ongoing energy consumption necessary for maintaining long-term data storage. The large-scale nature of medical data, especially genomic information, demands systems that can scale effectively, which most hospital-based systems struggle to achieve. This lack of scalability leads to inefficiencies that become more pronounced as data volumes grow, posing a serious challenge to the viability of in-hospital DNA storage solutions. In addition, the lack of integration between various technologies—ranging from data coding and DNA synthesis to storage, retrieval, and data security systems—further complicates in-hospital storage efforts. Without a cohesive framework, hospitals face fragmented, inefficient processes, which not only drive up costs but also create gaps in data accessibility and security. These issues highlight the urgent need for a more centralized, holistic approach to data storage. RDSCs offer a viable solution to these challenges by centralizing DNA storage for multiple hospitals in a given region, significantly reducing the strain on individual facilities. By consolidating storage functions in a few strategically located centers, the costs associated with maintaining and upgrading storage systems can be shared across a broader base of institutions, which in turn reduces the financial burden on each individual hospital. This setup allows hospitals to avoid the high capital and operational costs of maintaining their own storage systems, while still benefiting from state-of-the-art infrastructure that is regularly updated and optimized for long-term data preservation. The economic benefits of RDSCs extend beyond just cost-sharing. By situating these centers in sparsely populated areas, they can take advantage of lower operational costs, such as cheaper energy, lower land costs, and more scalable infrastructure. These advantages make RDSCs far more cost-effective compared to decentralized, in-hospital solutions, where energy costs and space limitations often escalate. Furthermore, the geographical concentration of storage systems allows for better resource allocation and enhanced operational efficiency. With secure internet connections linking RDSCs to multiple hospitals, medical cold data can be accessed safely and seamlessly across different institutions, without the need for each hospital to independently manage its own storage systems. This collaborative approach also fosters data exchange and standardization, which are crucial for advancing medical research and improving healthcare outcomes. By consolidating resources, improving operational efficiency, and enhancing data accessibility, RDSCs promise to address the critical challenges of cost, scalability, and sustainability in medical cold data storage.

Within this general framework, established technologies such as traditional four-base coding algorithms, DNA chemical synthesis, NGS, and PCR will continue to play a pivotal role. Although artificial nucleotides and DNA nanostructures hold significant promise in addressing certain limitations of DNA storage systems, they remain far from being practical for widespread use in the near future. Additionally, compared to the traditional four-base DNA storage system, systems utilizing artificial nucleotides or DNA nanostructures would require entirely new technologies for writing, accessing, and reading data. This added complexity makes these advanced methods harder to implement on a large scale in the immediate term. As a result, the traditional four-base coding system remain a highly viable solution for the RDSCs in near future. At RDSCs, medical data would be processed using the established DNA storage workflow that leverages current state-of-the-art technologies. While contemporary universal coding algorithms typically achieve similar coding densities of around 2 bits/nt, no single algorithm stands out as distinctly superior for medical cold data. Given the long-term retention needs of such data, a highly stable and accurate coding algorithm, such as the Fountain code or Yin-Yang code, would be preferred. These algorithms offer robustness and error correction capabilities, essential for ensuring the integrity of medical data stored over extended periods. However, given that innovations in coding do not escalate the costs of existing systems, developing specialized end-to-end coding algorithms tailored for medical cold data is an optimal approach, as it can improve data compression and storage efficiency without introducing prohibitive costs. Once encoded, medical cold data would be written, accessed, and read using well-established and cost-effective commercial technologies, including DNA chemical synthesis, NGS, and PCR. Although novel advancements in synthesis, sequencing, and access methods show promise in addressing some of the current DNA storage system limitations, most of these developments remain in the research phase and are not yet suitable for practical application. In the meantime, refining existing technologies, such as improving DNA block assembly for synthesis and redesigning primers for more efficient PCR-based data access, would likely enhance the performance and cost-effectiveness of DNA storage systems in the near term. Following encoding and synthesis, the data-embedded DNA molecules would be stored for long-term archival purposes. In this context, *in vitro* storage methods are generally considered more viable for medical cold data due to their durability, flexibility, and cost-effectiveness. Techniques such as silicon-based encapsulation and mixed drying with alkaline salts are particularly recommended. While mixed drying with alkaline salts may result in a slightly shorter storage duration, this approach remains advantageous due to its simplicity in handling and its ability to preserve DNA for over 100 years, making it an optimal solution for long-term data storage in the medical sector. As for *in vivo* DNA storage methods, their high costs and the complexity of manipulation currently make them less feasible for short-term application. However, in the long run, the development of *in vivo* DNA storage could address several critical limitations, providing a unique advantage by harnessing the self-repair, amplification, and low-cost maintenance properties of DNA within living cells. This could potentially offer a scalable and sustainable option for medical data storage, especially as advancements in gene editing and cellular technology continue to reduce costs and enhance the efficiency of *in vivo* systems.

While the establishment of RDSCs for medical cold data is technically feasible, it introduces significant challenges, particularly in the areas of technology integration, cost considerations, and data security. The most pressing issue lies in the integration of various technologies, both internally within the storage center and externally with existing hospital systems. Internally, despite rapid advancements, DNA storage technologies span multiple fields, including biology, information technology, and engineering, creating a fragmented landscape. Establishing and maintaining a DNA storage center requires a multidisciplinary approach that facilitates knowledge exchange and collaboration across these fields. To effectively manage the diversity and fast-paced evolution of the technologies involved in DNA storage, the workflow should be organized into distinct modules. This modular approach allows individual modules to be updated and optimized independently while maintaining compatibility with the overall system. For instance, when a new DNA synthesis technology becomes available, it can be integrated by simply replacing the synthesis module without the need for overhauling the entire system. This modular design also allows different modules to be located at different sites, enhancing feasibility. For example, the information department can be positioned within or near hospitals to ensure rapid response to data exchange and coding issues, while the DNA manipulation workshop and preservation warehouse can be placed in remote, environmentally adaptable locations to reduce resource consumption. Moreover, this distributed setup encourages collaboration between companies from various industries, fostering innovation and improving the practicality of the DNA storage system. In this context, the DNA storage solution based on modulation encoding and decoding developed by Liu's team serves as a modular example [161]. This architecture divides the data storage process into independent modules, such as modulation encoding and decoding, allowing for individual updates without affecting the overall system. Externally, the integration of the DNA storage center with existing hospital information systems is critical to ensuring smooth data transfer and management. Medical data comes in a variety of formats, including text, images, videos, genomic sequences, and more. Even within the same data types, variations often exist depending on the facility or equipment used. Establishing uniform standards for data formatting and standardization is essential for ensuring efficient and consistent DNA data storage. While it is technically possible to encode all medical data into DNA sequences using a single coding method, this approach is inefficient due to the complexity and variability of medical data. Rather than attempting to unify all hardware and software across hospitals, the focus should be on preprocessing medical data to conform to a format suitable for DNA encoding. The development of standardized protocols for medical data formatting is crucial for streamlining the data flow between hospitals and RDSCs. Specialized coding algorithms tailored to each type of medical data, such as genomic data, medical images, or clinical records, would facilitate this process, ensuring accurate and efficient data conversion. Given the large volume and high degree of similarity in medical data, these data-type-specific protocols would enhance the efficiency and scalability of the DNA storage system. Moreover, a uniform protocol issued by relevant authorities could help guide this development, fostering consistency and standardization across the healthcare sector and accelerating the adoption of DNA-based medical data storage.

The high cost of DNA storage represents a significant barrier that requires careful consideration, particularly in its current stage of development. The initial investment in DNA storage technology is substantially higher than that of traditional electronic storage systems. One of the primary cost drivers is DNA chemical synthesis, the process of physically constructing DNA strands based on digital data. While there have been steady reductions in the cost of DNA synthesis over recent years, it remains orders of magnitude more expensive than the cost of manufacturing traditional storage media. When considering the entire storage system, including reading, writing, and maintaining the infrastructure, the cost gap becomes even more pronounced, making DNA storage appear prohibitively expensive for widespread use. However, it is crucial to view this situation with a long-term perspective. DNA storage for medical cold data is intended for long-term retention. This longevity means that while the initial investment is high, the cost of daily maintenance and data management will be amortized over an extended period of use, significantly reducing the effective annual cost of the system. As DNA storage systems mature and technology advances, innovations in DNA synthesis methods, improved sequencing technologies, and optimized storage protocols are expected to lead to rapid reductions in cost. For example, improvements in enzymatic DNA synthesis and PCR-based methods for reading and accessing stored data could drastically cut costs, making DNA storage increasingly competitive with traditional storage solutions. Furthermore, the constant evolution of DNA storage related technologies will likely result in ongoing updates and optimizations, allowing the system to become more cost-efficient over time. Advances in automation, microfluidics, and high-throughput DNA sequencing platforms are likely to improve the scalability and efficiency of DNA storage, further driving down costs. Therefore, while the current cost of DNA storage for medical cold data remains high, its long-term potential should not be underestimated. As the technology evolves and becomes more widespread, the initial investment will be balanced by the system's ability to provide secure, durable, and highly efficient data storage for many decades. Additionally, the growing demand for long-term data preservation, particularly in fields such as healthcare, could provide the necessary market incentives for continued innovation and cost reduction, making DNA storage a viable and sustainable solution for the future.

In the context of medical data storage, ensuring the security and integrity of the data is of paramount importance. Medical data is highly sensitive and protected by strict confidentiality laws. Therefore, comprehensive lifecycle management of DNA-based medical archives must address the interconnected challenges of preservation, replication, deletion, and transmission, each of which plays a crucial role in maintaining data integrity and confidentiality. Maintaining the integrity of DNA data over extended periods is a key challenge in DNA storage, especially for medical data that must be preserved for decades or longer. Optimal preservation conditions are crucial to ensure the longevity and stability of the encoded information. This involves controlling various environmental factors such as temperature, humidity, and exposure to UV light or contaminants, all of which can degrade DNA and compromise the stored data. Special care must be taken to maintain these factors within precise limits to prevent any chemical breakdown or physical damage to the DNA molecules. To mitigate the risk of degradation, *in vitro* preservation techniques such as silicon-based encapsulation and mixed drying with alkaline salts have proven to be effective. These methods provide a stable environment for DNA, protecting it from external environmental factors that could lead to data loss. While encapsulation methods provide excellent protection, ensuring that the DNA is stored under optimal conditions is equally important, especially when considering long-term medical data retention that exceeds 100 years. Regular monitoring of environmental conditions is necessary, and automated systems for maintaining these conditions could be integrated into the DNA storage infrastructure to ensure consistency over time. Despite all precautions, emergency data recovery plans remain essential to address unforeseen data loss or corruption, ensuring that critical medical data can still be retrieved if needed. Data replication is a critical component of maintaining data integrity in DNA storage systems. DNA-based storage systems often rely on redundancy to mitigate errors during DNA synthesis and sequencing. One widely used technique is PCR amplification with degenerate primers, which generates 8–12 redundant copies of each file. This redundancy helps ensure that even if some copies of the data are corrupted or degraded, other copies can be used to recover the original information. Moreover, ECCs, such as Reed-Solomon codes, are integrated into the DNA storage process to further reduce the possibility of errors. These codes help to correct sequencing errors and ensure that data remains intact even if some of the DNA strands experience slight degradation. By using ECCs, the system can suppress sequencing errors to levels below 10^9^, ensuring that the integrity of the data is upheld even in the case of minor errors during the reading process. This level of replication and error correction makes DNA storage systems highly reliable for medical data that require long-term retention and precision, even when dealing with large volumes of data. Data deletion is another crucial aspect of managing DNA-stored medical data, especially in terms of compliance with regulations and privacy laws. Since medical cold data often involves bulk erasure, simple, cost-effective methods for deleting DNA data are needed. UV irradiation (10 min at 265 nm) and DNase I digestion are currently the most practical and economical techniques for erasing data, with costs around 0.08USD/GB. These methods break down the DNA to prevent any recovery of the data, ensuring that sensitive information is completely destroyed when no longer needed. For more precise, targeted deletion, CRISPR-based systems offer a potential solution. These systems allow for the specific removal of selected data strands, but their operational cost is significantly higher, making them impractical for routine use in large-scale DNA storage systems. Nonetheless, the availability of both methods allows for flexibility in managing data security, ensuring that medical data can be deleted in a way that aligns with regulatory and security requirements. Transmission of medical data stored in DNA form between institutions or RDSCs must be secure and efficient, as the confidentiality and integrity of the data must be maintained throughout the transfer process. Encrypted SSL/TLS channels are currently the primary method used to transmit DNA sequence files between medical centers and storage facilities. These encrypted channels ensure that data is protected from unauthorized access during transmission, adhering to standard data security practices for medical information. Emerging technologies, such as microfluidic automation and blockchain-enhanced verification, are showing potential for further strengthening data transmission security. Microfluidic automation can automate the handling and transfer of DNA-encoded data, reducing the risk of human error and ensuring that the DNA molecules are processed correctly before being transmitted. Blockchain technology can be used to verify the integrity of the data being transferred, providing an immutable record of data exchanges and ensuring that the data has not been tampered with during transmission. Access control during transmission is also critical. Medical data should be transferred under strict safeguards, with only authorized personnel able to initiate and complete data exchanges. This can be achieved through robust authentication and authorization mechanisms, ensuring that only individuals with the proper credentials can access or transmit the data. Additionally, the use of DNA-specific encryption methods that leverage the unique properties of DNA molecules could provide an added layer of security, making it more difficult for unauthorized parties to intercept or decode sensitive medical data during transmission.

## 5 Summary and outlook

The digital revolution has marked the beginning of the information age, fundamentally altering the relationship between humans and data. The increasing diversity and volume of data, especially in healthcare, pose significant challenges to current storage capabilities. To address the demands of large-scale data storage, innovative solutions that offer enhanced efficiency and reduced costs are essential. DNA storage, capitalizing on the unique biological properties of DNA, presents itself as a promising alternative to traditional storage media.

Recently, DNA storage technology has attracted widespread attention and significant investment from leading companies such as Microsoft, Illumina, BGI, and prestigious universities like the University of Washington and the University of Cambridge. This increase in interest and investment in research highlights the growing potential for the applications of DNA storage.

Researchers have reviewed DNA storage technology and predicted that with advancements in synthetic biology, microfluidics technology, computer science, bioengineering, and materials science, new technologies such as microfluidics designed specifically for DNA data storage, DNA enzymatic synthesis and nanopore sequencing will improve automation and integration, accelerate synthesis and readout efficiency, and reduce cost. Additionally, the development of more efficient coding and error-corretion algorithms will increase data storage density and information integrity. These technological advancements will make DNA storage increasingly economically viable and eventually lead to breakthroughs in practical applications [[162], [163], [164]].

However, it will possiblely take at least several decades.

In this review, we concentrate on evaluating current DNA storage workflow technologies and designing a DNA storage system tailored for medical cold data storage in the near future. We propose the establishment of RDSCs servicing multiple hospitals as a pragmatic approach. Our projections underscore the immediacy and feasibility of developing high-density coding methods tailored to medical cold data. Well-established commercial technologies, such as DNA chemical synthesis, NGS, mixed drying with alkaline salts, and refined PCR, are regarded as the optimal methods for data writing, reading, storage, and accessing, respectively. Data security would be based on not only proper physical protection but also the combination of traditional digital encryption and DNA steganography. Although advancements like artificial nucleotides and DNA nanostructures, together with innovative DNA synthesis, DNA sequencing, and data accessing methods, show considerable promise, they still reside in the research phase and are unlikely to be realized in the near future.

Nowadays, the storage of DNA samples from patients is already clinically available. DNA data related to cancer diagnosis and treatment, genomics research, and genetic diseases testing are already stored using current technologies. Therefore, RDSCs are technically feasible at present. However, they still face the challenges of technology integration, data integrity, and, most importantly, high cost. Before the costs of DNA synthesis and sequencing decrease significantly, it is unrealistic to convert extensive patient records into DNA, despite the technology's high storage density and longevity making it an attractive option for the near future.

To address the aforementioned challenges, future directions for the application of DNA storage will include: 1) Further reducing the costs and enhancing the speed of DNA synthesis and sequencing, 2) Developing efficient and high-density coding methods, 3) Enhancing random access capabilities, 4) Training a professional team dedicated to routine maintenance and troubleshooting, 5) Integrating multi-level technologies, encompassing both internal workflows and external systems.

In conclusion, DNA storage presents itself as a promising pathway for the medical cold data storage. In the short term, *in vitro* DNA storage stands as a viable option, providing feasibility, scalability, and stability. Although *in vivo* DNA storage addresses certain limitations of *in vitro* storage, it is still in the nascent stages of development. However, beyond data storage, *in vivo* DNA storage technology can also function as a biological recorder or gene-editing tool, with profound implications for biomedical research and applications. Therefore, investments *in vivo* DNA storage research and development could transcend commercial interests to achieve world-class research status and, in the long-term, potentially outpace *in vitro* DNA storage.

## Funding statement

None.

## CRediT authorship contribution statement

**Peilin Shen:** Writing – original draft, Methodology, Investigation. **Yukui Zheng:** Methodology, Investigation. **CongYu Zhang:** Writing – review & editing. **Shuo Li:** Investigation. **Yongru Chen:** Investigation. **Yongsong Chen:** Supervision. **Yuchen Liu:** Supervision. **Zhiming Cai:** Project administration, Conceptualization.

## Declaration of competing interest

The authors declare that they have no known competing financial interests or personal relationships that could have appeared to influence the work reported in this paper.
